# Benefits and challenges: Qualitative exploration of women’s experiences during the COVID-19 pandemic in Fiji

**DOI:** 10.1371/journal.pone.0331794

**Published:** 2025-09-04

**Authors:** Eunice Okyere, Kissinger Marfoh, Ditoga Kabukeinamala, Ramneek Goundar, Litia Makutu, Latileta Odrovakavula, Sovaia Vasukinatavea

**Affiliations:** 1 Department of Public Health, College of Medicine, Nursing and Health Sciences, Fiji National University, Suva, Fiji; 2 Department of Epidemiology and Environmental Health, College of Medicine, Nursing and Health Sciences, Fiji National University, Suva, Fiji; Group for Technical Assistance / Asian College for Advance Studies, Purbanchal University, NEPAL

## Abstract

The COVID-19 pandemic and its preventive strategies resulted in changes in economic, social and health activities globally, but the effects of these changes on women, have not been systematically studied and documented in Fiji. The current study explored the experiences of women during the COVID-19 pandemic in Fiji, using a qualitative approach with a descriptive phenomenological design. A total of 110 women were purposively selected across various age groups, ethnicity, religion and different settings to improve the study area diversity. In-depth interviews were conducted using a semi-structured interview guide, and analyzed inductively, using the thematic approach. The three themes that emerged were employment and financial issues, health challenges during the COVID-19 pandemic and COVID-19 social and health benefits. The employment and financial issues identified included fear of losing job, difficulty finding a new job, job renewal challenges, decline in private businesses and high cost of living. Participants experienced health challenges including overweight and obesity, fear and anxiety, depression, insomnia and feeling helpless and worsened pre-existing health conditions. The COVID-19 social and health benefits included high sense of responsibility towards extended families, strengthened core family relationships, work-life balance, improvement in health status, healthy lifestyle behavior, and self-care awareness. The findings indicate the need for employers to address the employment and financial needs of women during emergency situations, like the COVID-19 pandemic. Health care managers, health care workers and policy makers should implement strategies to address the COVID-19 health challenges and strengthen the COVID-19 social and health benefits to improve the health of women, in the study area.

## Introduction

The COVID-19 pandemic created upheaval in nations around the world. As such, various countries, including Fiji Island, instituted different preventive strategies to reduce the spread of the virus, which resulted in disruptions in economic, social and health related activities [1–3]. Restrictions such as social distancing, self-isolation, and remote working, contributed to reducing the spread of the virus with severe economic implications [4]. These measures affected the global travel business, global trade, and financial status of individuals, due to the collapse of many supply chains and daily business activities. Spillover effects to developing countries were expected due to their dependence on developed countries for economic support [5,6].

The uprising economic crisis prompted serious issues and challenges in relation to socio-economic determinants of health [7]. Previous studies have identified economic uncertainty as a high-risk for the overall health and well-being of individuals and their relatives [7,8]. Decline in economic activities could lead to cognitive and affective disturbances from the complex interplay of many devastating factors, including unemployment, downsizing of social services, and reduced public spending [9–11]. Past experiences on financial crisis, offer understanding into potential problematic points and knowledge about possible risks, with economic uncertainties, regarded as an important component [12,13]. Uncertainty of the future, lay-offs and loss of jobs, including private businesses have led to severe mental conditions [14], worsening existing health conditions [15,16].

Employment loss slows down career development, especially for women and affects their future earnings. It is expected that, many women who have lost their jobs during the COVID-19 pandemic will experience a significant drop in future incomes [17]. Women earn less, and accrue less wealth than men [18,19] but men’s livelihoods have improved more quickly than women after previous pandemics [20]. As such, the COVID-19 pandemic is likely to affect the prospect of women in the labor market including their earnings and development compared to men [21,22], subsequently, leading to an increased in gender inequality [23]. Other scholars have highlighted the challenges women faced during the pandemic in relation to increased responsibility in family care and domestic duties. These researchers revealed that, irrespective of women’s employment status, they still spend much time to cater for their family members and other household duties compared to men [24,25].

In the Pacific Island Countries, noncommunicable diseases such as diabetes, cardiovascular diseases, cancer and chronic respiratory diseases, contribute to the largest cause of premature deaths [26]. Pacific Islands countries including Fiji, account for 9 out of 10 of the top countries in the world with the highest prevalence of diabetes and obesity among women aged 20 and above [27]. In Fiji, 30% of the population has diabetes, with diabetic foot amputations increased from 1 every 12 hours to now 1 every 8 hours [28,29]. Fiji Islands is an archipelago comprising of more than 300 Islands covering over 18,000 square kilometers. The geographical position presents large gap in the availability and delivery of health services, to a diverse population over a large maritime region, with the situation worsened by the impacts of climate change and natural disasters [30,31]. The COVID-19 pandemic could have compounded the situation, hence the importance of this study.

Though the COVID-19 pandemic tested the social fabric of all countries, the impact could be more in Fiji, given that the communities are largely communal and conservative [1,32]. The inability of individuals, particularly women, to associate with relatives and friends, could cause not only stress-induced mental conditions harmful to psychological well-being but also identity disturbances, where the population is confronted with inconsistencies related to their perceived subjective identities and the new emerged reality created by the COVID-19 pandemic [33]. The pandemic disrupted the normal flow of the society, and some people might find it difficult to readjust, subsequently, leading to various health related issues such as anxiety, depression, and stress [34,35]. Studies have attributed these health outcomes to pre-existing health conditions, home-schooling, financial difficulties, and future uncertainties [36]. Combined with compulsory quarantine and social isolation in various countries worldwide, these factors had impacted the general health and well-being of individuals, including women [37,38].

Women in various countries were not equally affected by the crisis posed by the COVID-19 pandemic. Therefore, the relevance of this study, undertaken in the Fiji Island, to explore women’s experiences, particularly within the context of the pandemic. Most of the past studies that examined COVID-19 effects on women, used quantitative approach and were conducted in developed countries like Canada, United States, and Europe. These studies mainly identified mental health issues such as depression, anxiety, and stress [39–43]. Our study aims to expand past research by using qualitative approach to explore and understand employment and financial issues, health challenges and the social and health benefits of the COVID-19 pandemic, among women in Fiji. Conducting this study in the Fiji society with high cultural values and dominant patriarchy nature [44], distinguish this study from other previous studies and provides baseline information for further research. This study therefore seeks to answer the following questions:

(1) What are the employment and financial issues faced by women during the COVID-19 pandemic?
(2) What are the health challenges faced by women during the COVID-19 pandemic?
(3) What are the health and social benefits experienced by women during the COVID-19?

The knowledge from this study could inform employers and policy makers about issues women encountered in relation to their jobs and finances, for appropriate interventions. This research also contributes to literatures in behavioral science and health, by highlighting essential information that health managers, health workers, and other relevant stakeholders can use to lessen the short- and long-term undesirable effects of the COVID-19 pandemic on women’s health. Additional contribution includes shedding light on the COVID-19 social and health benefits to reinforce positive health behaviors, among women, in the study area.

## Methods

### Study design

A qualitative approach with a descriptive phenomenology design was used to explore and understand the experiences of women during the COVID-19 pandemic. Particularly, participants were requested to share their opinions on employment issues, health challenges and social and health benefits. Qualitative approach enables researchers to investigate real-life situations in detail, offering a deeper understanding of the phenomenon or occurrence understudy [45,46]. The flexibility and adaptability nature of this method enabled the researchers to obtain deeper, detailed, and richer data from the women, in the study area [47].

Descriptive phenomenology focuses on individuals’ perceptions and experiences on a particular phenomenon, and this should be free of assumption or theory to enable phenomenological reduction [48]. The researchers considered the phenomenological reduction process to collect and analyze the data, by putting aside all judgements or beliefs about the external world and taking nothing for granted in everyday reality [49]. This design enabled the researchers to examine the lived experiences of women during the COVID-19 pandemic. Apart from the collective experiences of the pandemic, individual experiences also existed, and everyone’s experience could be influenced by their life circumstances. The objectivity associated with the pandemic is entangled with the subjectiveness of the individual living in the pandemic. Therefore, the individual experiences of these women formed the actual source of knowledge in this research [50].

To enhance rigor in the methods used, this study took into consideration the concept of reflexivity. By so doing, the researchers acknowledged the role they play in knowledge creation and recognized that meanings are conveyed within a particular occurrence, context, and settings [51,52]. Reflexive notes were taken during the in-depth interviews which provided in-depth information to enrich the data collected [53]. The researchers maintained a reflective attitude throughout the entire research process by questioning the understanding of data and themes obtained [54].

### Study setting

This research was conducted among 110 women in Suva and its bordering surburbs including Lami, Nausori and Nasinu. Selecting participants from across these areas and from different age groups, ethnicity, religion, marital status and occupation, improved the study area diversity and enabled the researchers to get a deeper understanding of the topic under investigation [55]. Suva, the capital of Fiji, is the largest metropolitan city. It is situated on the southeastern coast of the island of Viti Levu, located in Rewa Province in the Central Division.

### Participants recruitments

Invitation letters describing the aims and the need for women to participate in this study were distributed to potential participants through direct engagement. Community sensitization was done through the community leaders, and this included house to house visits where relevant information on the study was provided to the study participants. Information sheets containing the study details were handed to them to enable them to decide to participate in the study [56]. Interested participants contacted the investigators who visited to determine their eligibility using the inclusion criteria which included, women living in the study area, 18 years and above and without mental disorder. The exclusion criteria were women residing outside the study area, had mental disorder and not willing to participate in the study.

Participants were selected purposively for this study, and this enabled the researchers to select respondents who can provide detailed and rich information on the phenomenon understudy. Purposive sampling relies on researchers’ discretion to select participants from the study population [57], but it is recommended for researchers to be informative about the purpose of the study, taking into consideration, the inclusion criteria [58]. This enabled the researchers to explain the impact of the findings on the population.

### Data collection

In-depth interviews were conducted between September 2022 to February 2023, with the study participants’, using semi-structured interview guide (S1 File). The interviews were conducted by four (4) trained research assistants, who had no previous or ongoing personal relationship with the study participants. The research assistants were trained by experienced qualitative researchers (EO, LM, RG and KM). Each interview lasted for about 30−40 minutes and the average time of the interview was 38 minutes. The interviews were audio recorded to capture the exact words of participants [59]. Permission was sought from all interviewees before recording the interviews. The semi-structured interview guide was pre-tested among five women outside the study area to assess the content, structure, question orders, terms, and cognitive responses, and revised accordingly for the actual interviews. Assessing the feasibility, reliability and validity of the guide improved the data quality, thereby meeting the study objectives [60]. The face-to-face interviews were conducted at venues participants considered comfortable, with minimized noise and interruptions. Both the interviewer and the interviewee agreed and approved the time for the interview. The interview environment was suitable and provided comfort and privacy for participants to be able to discuss sensitive matters [35]. Relevant probes or follow-up questions were used to obtained rich information from respondents. Interview probes focused on the effects of the COVID-19 pandemic on women’s social life and livelihood, how their employment status was affected, the health issues they faced including access to health services, their experiences and the behavior change practices that occurred during the pandemic. The data collection was halted when data saturation was reached [61].

### Data analysis

The data were analyzed using the thematic analysis approach because it employs systematic process to manage qualitative data to improve the data structure and consistency, thereby improving trustworthy and insightful findings [62]. Thematic analysis has been identified as suitable to examine the perceptions and experiences of study participants highlighting similar and different views and generating unanticipated understandings on the phenomenon under study [63].

The research assistants transcribed the data verbatim. The data analysis began with two of the researchers (EO and DK) familiarizing themselves with various aspects of the research data. The researchers ensured that the transcripts provided a verbatim description of all verbal and non-verbal information [54]. For accuracy, the analysts checked and cross-referenced the transcripts against the actual audio-recordings [64]. The two authors independently read the transcripts in an active and repeated manner to familiarize themselves with the depth of the data content, in search of meanings and patterns. The analysts took notes and wrote down initial ideas for the actual coding [65]. In the second stage, the analysts generated initial codes, by organizing the data into meaningful groups. Thus, essential segments of the raw data were coded [66]. To enhance rigor and consistency, the analysts paid attention to each item in the data set and went deeper to search for interesting and meaningful parts of those items, using highlighters that finally created the basis for the formation of themes [67].

The next phase involved the searching of themes and reviewing those themes. The analysts began to search for themes after data have been coded and collated by focusing on themes at the broader level, which basically entailed grouping of codes to form overarching themes. The analysts used maps to help in categorizing the different codes into themes and searched for the interconnections and links between different codes and identifiable themes. While some codes led to the formation of the main themes, others formed sub-themes [66]. The emerging themes were then reviewed to ascertain whether data extracts fit into those specific themes, which led to the generation of a thematic map (S2 File). The researchers than checked to see if the generated map of themes was a true reflection of the meanings and essence of the entire data set [63]. This enabled the researchers to gain a detailed understanding of the different themes, how they linked with each other and the narratives they conveyed.

After the themes have been searched and reviewed, the researchers proceeded to define them by finding the ‘essence’ of each theme. They labelled each theme and identified the story each theme conveyed and how each of these stories fits into the overall narrative that the researchers wanted to communicate. For consistency, themes were considered both independently and in relation to other themes [68]. At this point, the researchers identified the themes and sub-themes of the study and described the content of each theme. Themes were finalized after the researchers had examined the coding carefully in a repeated manner [65].

The last stage of the analysis involved producing the final report, where the researchers communicated the story the data conveys in a logical, concise, and coherent manner, across the themes and sub-themes established [66]. Data extracts in the form of direct quotes from interviewees were used to demonstrate the essence of each theme and the story in general [63,66].

### Methodological rigor and trustworthiness

This study established rigor and trustworthiness by meeting the credibility, confirmability, dependability, and transferability criteria [69]. The methods used in the data collection improved credibility. This includes the use of the semi-structured interview guide, which allowed the researcher to use probes to obtain detailed and rich information on the phenomenon understudy. Probing is essential since it encourages study participants’ and direct them towards providing important, comprehensive, and genuine responses [70]. Recording the interviews using an audio-tape recorder ensured that the exact words of participants were captured to improve the quality of data collected [59].

The confirmability criterion was assessed through an audit trail, where the researchers provided a step-by-step procedure for conducting this study [71]. Effective communication was sustained among the researchers to ensure that those involved in collecting the data abided by the study protocols. The data collection team systematically reviewed the transcripts against the recorded interviews and the research team approved the steps used to code the data and identify the themes and sub-themes, for consistency and accuracy [72].

Monitoring the steps involved in the data analysis improved consistency and enabled the researchers to interpret the results correctly. The dependability criterion was measured by providing detailed description of this study processes which include the methods used to collect the data, conduct analysis, and interpret the findings [73]. For transferability criterion, as shown in the method sections, the purposive sampling used, enabled the researchers to select participants across different age groups, to improve the study area diversity [74]. The research team ensured that rigor, trustworthiness, and ethical processes were sustained during this study, to enhance the research quality.

### Ethical consideration

This study received ethics clearance from the Fiji Human Health Research Ethics Committee (FHHRERC), Ministry of Health and Medical Sciences with approval number, 18/2022. Written informed consent was obtained from all participants before starting each interview. Information sheet containing essential and detailed information on the study, was provided to the study participants, to enable them to obtain deeper understanding on the study. The information sheet included the study aim, methods, potential benefits, funding source and the institutional affiliations of the researchers. The researchers systematically addressed the concerns and questions from the study participants. Respondents were informed about ways to disseminate the findings of this study including publication.

Participants were made aware about the voluntary nature of the research. Thus, they have the right to withdraw from the study at any time or refuse to answer any question during the interview, and this will not affect them in anyway. To improve anonymity and confidentiality, respondents’ actual names were substituted with codes in the audio recordings and all identifiers removed from the data so that they will not be identified [75].

## Results

Majority of the 110 women interviewed were civil servants (52%), married (71%) and belonged to the I-Taukei ethnic group (50%). Most of them were Christians (56%) and lived in Nausori (29%). The age ranged from 26–62 years and most of the women had 4–6 children (53%). In this study, single women were those who had never been married, widowed or divorced (Table 1).

**Table 1:** Demographic characteristics of participants interviewed.

Study Participants (Women)	N = 110
**Median Age in years**	**44 (26-62)**
**Ethnicity**	
I-Taukei	55 (50%)
Indo-Fijian	46 (42%)
Others	9 (8%)
**Marital Status**	
Single	32 (29%)
Married	78 (71%)
**Religion**	
Christian	62 (56%)
Moslem	25 (23%)
Hinduism	20 (18%)
Others	3 (3%)
**Occupation**	
Civil servant	57 (52%)
Private sector	18 (16%)
Self-employed	23 (21%)
Unemployed	12 (11%)
**Study Settings**	
Suva	30 (27%)
Nausori	32 (29%)
Lami	28 (25%)
Nasinu	20 (18%)
**Number of children**	
1-3	36 (33%)
4-6	58 (53%)
7-9	16 (14%)

Inductive analysis of the data resulted in three broad themes and sub-themes. The themes included Employment and financial issues, Health challenges during the COVID-19 pandemic and COVID-19 social and health benefits (Table 2).

**Table 2:** Themes and Sub-themes.

Themes	Sub-themes	Codes generated	Repetition of codes
Employment and financial issues	Fear of losing job	unemployment anxieties, frequent job loss, job retention concerns, economic decline, sudden job loss, unpaid employees, unstable unemployment conditions, mass job losses, unemployment panics	frequent job loss, unemployment anxieties, mass job losses, sudden job loss, unemployment panics, economic decline,
	Difficulty finding a new job	lack of jobs, shortage of funds, global unemployment crisis, few jobs advertised, employment restrictions, savings depletion, economic crisis, few job options,	global unemployment crisis, lack of jobs, employment restrictions, few job options,
	Job renewal challenges	sudden job termination, demanding contract, complex job conditions, learning difficulties, compulsory professional development, limited career pathways, unfair contract terms, expensive job training,	demanding contract, compulsory professional development, unfair contract terms, expensive job training, learning difficulties,
	Decline in private businesses.	low purchasing power, sale loss, lack of support, small business closure, loss of customers, low profit, sales decrease, reduced economic activities, low income	small business closure, low purchasing power, reduced economic activities, sales decrease, low profit,
	High cost of living	increased food prices, unstable market conditions, increased transport cost, low salary, high items cost, financial struggles,	increased food prices, financial struggles, unstable market conditions, increased items cost,
Health challenges during the COVID-19 pandemic.	Overweight and obesity	excessive weight gain, overeating, uncontrolled weight increase, difficult reducing weight, unhealthy weight diagnosis, emotional eating, lack of exercise, high sugar diets, no weight checks	uncontrolled weight increase, emotional eating, unhealthy weight diagnosis, difficult reducing weight, overeating, excessive weight gain,
	Fear & anxiety	fear of covid-19, stressful feelings, panic attacks, continuous sadness, feeling lonely, state of confusion, covid-19 mass death, social media misinformation, fear of death, excessive worry, vaccine mistrust, fear of uncertainties,	panic attacks, fear of covid-19, fear of uncertainties, stressful feelings, state of confusion, feeling lonely, excessive worry, continuous sadness,
	Depression	overthinking, emotional distress, unexpected family deaths, persistent grief, miscarriage panics, job loss frustrations, emotional exhaustion, feeling of guilt, relationship conflicts, prolong mood swings, relationship breakdown, ineffective communication,	miscarriage panics, persistent grief, unexpected family deaths, emotional distress, relationship conflicts, feeling of guilt, relationship breakdown, overthinking, emotional exhaustion,
	Insomnia and feeling helpless	life uncertainties, low income, financial constraint, lack of support, severe sleep deprivation, irregular sleep pattern, tired of life, feeling abandoned, covid-19 misinformation, job loss concerns	severe sleep deprivation, life uncertainties, covid-19 misinformation, feeling abandoned, job loss concerns, irregular sleeping pattern, lack of support,
	Worsened pre-existing health conditions	foot amputations, hypertension, diabetes complications, high glucose level, health workers neglect, lack of medications, poor eating patterns, inadequate physical activities, herbal medications use, chest pains, constant anxiety state,	high glucose level, foot amputations, health workers neglect, hypertension, herbal medications use, chest pains, poor eating patterns, diabetic complications.
COVID-19 social and health benefits	Improvement in health status	eating healthy foods, exercising, routine glucose checks, regular medication intake, strict dietary intake, following health directives, blood pressure checks, maintaining blood sugar	strict dietary intake, regular medication intake, following health directives, exercising, eating healthy foods, maintaining blood sugar, blood pressure checks,
	Self-care awareness	following covid protocol, increased vegetable intake, prolonging lifespan, eating more fruits, death prevention, avoiding diabetic complications, supporting family, covid-19 prevention, health consciousness, weight loss, boosting immune system, reduced carbohydrate intake	avoiding diabetic complications, eating more fruits, following covid-19 protocol, increased vegetable intake, death prevention, supporting family, boosting immune system,
	High sense of responsibility towards extended families.	encouraging relatives, fostering effective communication, providing financial support, sharing food items, promoting family togetherness, fulfilling family obligations, protecting relatives, minimizing guilt, promoting family culture,	promoting family togetherness, protecting relatives, encouraging relatives, providing financial support, minimizing guilt, fulfilling family obligations,
	Strengthened core family relationships.	spousal companionship, quality family time, children availability, increased family bond, effective communication, family support, sharing responsibilities, spouse availability,	spouse availability, effective communication, children availability, spousal companionship, quality family time, increased family bond,
	Work-life balance.	work flexibility, taking work breaks, enjoying family presence, reduced workload, family collaboration, increased family time, efficient task management, staying organized, minimizing distractions,	reduced workload, work flexibility, increased family time, enjoying family presence, taking work breaks,
	healthy lifestyle behavior	avoiding alcohol, managing diabetes, avoiding smoking, following health directives, exercising	avoiding smoking, following health directives, managing diabetes, avoiding alcohol,

Presented below are the findings under the themes and sub-themes together with explanatory statements from the women interviewed.

### Employment and financial issues

#### Fear of losing job.

Participants were afraid of losing their jobs due to the economic crisis created by the COVID-19 pandemic. Most of them expressed concerns about the possibility of maintaining their jobs since some of their family members and friends had lost their jobs during the pandemic.

“To be frank my biggest fear is losing my job since this has become the order of the day. Even two of my cousins and some friends have lost their jobs because of COVID-19. Our economy is going down, so anything is possible”. (W4)

“I am worried because I think from the way things are going, my employers can terminate my job at any time. The last time, they sacked almost all the cleaners and brought new ones so it can happen to me too”. (W2).

#### Difficulty finding a new job.

Participants who had lost their jobs found it difficult to get new jobs, given the higher level of unemployment, resulting from the pandemic. As such, many depended on their savings for sustenance, subsequently, depleting their savings. However, low salary earners, especially single mothers, didn’t have enough savings to cushion them, after losing their jobs, which worsened their conditions.

“I lost my job because of COVID, and I have started looking for a job that can fetch me some money to sustain myself and my family, but it seems that is not possible now. I saved some money from my previous work, but that money is almost finished because I used some to buy food, pay rent and pay bills. It is a big problem but there is nothing I can do”. (W 7)

“I am sad…I have lost my job, and I don’t have any money saved in my bank account to cater for my children. I could not save any money because my salary was not even up to 1000 dollars a month and I have children to cater for. I am a single mother with 6 children so you can imagine what I am going through financially”. (W70)

#### Job renewal challenges.

Some of the respondents mentioned the difficulties involved in fulfilling some conditions for the renewal of their job contracts. This is in accordance with some employers’ policies that mandate staff with lower qualifications to upgrade their skills, to maintain their jobs. While few of the women found it difficult to concentrate on their studies during the pandemic, others complained of limited money to pay for their fees, to fulfill this mandate.

“…I have received a letter from HR [human resource] to upgrade my qualification, else I will lose my job but it’s not easy to learn and go through COVID-19 stress at the same time. I have enrolled for some online courses to upgrade my qualification and keep my job, but I cannot concentrate”. (W 10)

“I must upgrade myself to maintain my job, but the problem is, nobody is paying the school fees. I have to pay my fees from my salary, which is not enough. The best thing for me to do now is to look for a new job but I don’t think I will get one because COVID-19 has affected the economy. I have no option than to upgrade my skills to keep my job”. (W 15).

#### Decline in private businesses.

Many of the participants who initially engaged in private businesses, such as, selling of goods and farming to supplement their salaries, expressed difficulties in indulging in such activities during the COVID-19 pandemic, which led to low income.

“I am not a government worker. I buy fish from fishermen and sell in small quantities in the market, but everything has come to an end because of COVID-19. Fishermen have been banned from going to the sea to fish, so the business has gone down. I know the little money I have will soon finish because things are expensive now”. (W90)

“I was making a lot of money from sewing. I have worked as a seamstress all my life, but I have to close my shop because of COVID-19. My sewing business is going down every day because people don’t have money to sew new dresses. Only few family members bring their materials for sewing and they don’t even pay for the sewing because they don’t have money. It’s a big problem but I pray things get better soon”. (W85)

#### High cost of living.

Majority of the participants mentioned the increased in prices of items as one of the major challenges during the pandemic. Despite the high cost of living, their salaries remained the same, making it difficult for them to save money and life comfortably.

“In fact, the prices of things have gone up. Even food is expensive because of COVID-19…at first, I was able to save some money from my monthly salary but now I cannot do that because things are very expensive, but the government has not increased my salary.” (W19).

The employment and financial issues identified among women, included fear of losing their jobs, job renewal challenges, and difficulty finding new jobs. Participants losing their jobs including their private businesses and the high cost of living, led to low income and depletion of their savings.

### Health challenges during the COVID-19 Pandemic

#### Overweight and obesity.

Majority of the participants gained weight during the COVID-19 pandemic because they used food as a coping mechanism to reduce stress and anxiety resulting from the pandemic. According to the participants, the pandemic pushed them to eat more food than necessary, leading to overweight and obesity.

“COVID-19 did not help at all because I was eating too much, and I have put on too much weight during the lockdown. I have become obese, and I think I need to start checking my weight now because I don’t want to get diabetes and other diseases.” (W2)

“I was slim before the pandemic, but I have now become big because I eat a lot. My problem is I eat to relax and to take my mind off COVID-19 issues because the anxiety is too much. Anytime I get scared, I find some food to eat and become happy. This is what is keeping me alive but I’m gaining too much weight. Last week, I felt some heaviness in my check and when I went to the hospital, the nurse told me to reduce my weight because I am overweight. I don’t want to become obese [obesity] and get diabetes and hypertension like my other family members”. (W74)

#### Fear and anxiety.

One of the main concerns of the study participants was the fear of getting sick or dying from COVID-19. These mental and emotional distress were heightened by the frequent exposure to news from social media, including the large number of people dying from the disease.

“We are all scared of death, so COVID-19 is a problem for everybody. The cases are going down, but I am still afraid because anything can happen to anybody at any time. I have taken the vaccine, but I don’t think it can help so I am scared and feel stressed every day thinking about this pandemic”. (W32).

Many of the women who had experienced isolation expressed high levels of anxiety, confusion, and stress. According to these women, isolation or quarantine increased their state of loneliness and frustrations and worsened their mental health conditions.

“My mental health state became worse after quarantine because I felt lonely in my isolation room and became too anxious that anything can happen to me. Hearing about COVID-19 deaths stressed me and made me confused. I don’t even know if I can get back to my normal mental state”. (W82)

#### Insomnia and feeling helpless.

Participants found it difficult to sleep because of COVID-19 and felt helpless because they believed nothing could be done to improve the situation. While some attributed their inability to sleep soundly to misinformation and misleading rumors of the pandemic, others linked this to limited income, due to job loss. Accordingly, respondents explained their situation during the in-depth interviews.

“…To tell you the truth, I am not able to sleep at all because of COVID-19 pandemic. COVID-19 information is very scary, and we know some are not true, but I can’t stop thinking about them. The media reports new things every day and this has affected my sleep badly. Do you know that my BP [blood pressure] has gone up so I am taking BP medications now? (W90)

“I feel everything has come to an end for me because I have lost my job, and I don’t have enough money to even feed my children. I don’t think anybody can help in this situation because everybody is fighting COVID-19 problems. I stay awake every day thinking and it’s affecting my health”. (W15)

#### Depression.

Some of the participants were grieving because of loss of family members, subsequently, leading to a state of depression. Many of those who had lost their family members experienced a sense of guilt from their inability to protect their families. They thought they didn’t do enough to protect or prevent them from dying.

“I went through a state of depression, and I am still fighting with it, because I lost one of my immediate family members because of COVID. There is no way you can stop mourning over losing a family member so anytime you remember, it will push you into a state of depression and fear”. (W1)

“COVID-19 has made me depressed, and I am now taking medications because my dad died in another town, and I couldn’t even see his dead body. My two uncles also died from the pandemic, and I couldn’t attend their funerals. I always feel so guilty because I feel I didn’t protect them enough and that is why they died. I don’t think I can ever forget this in my life”. (W44).

Others attributed their state of depression to uncertainty about the future and relationship conflicts, resulting from the lockdown.

“… truth is I have gone through too much depression because of the pandemic. All my plans have come to a halt, and I don’t know what the future will bring to me. Another thing is that my partner has been away from me due to the lockdown, and we were fighting on phone every day and you know this is depressing. Now we are divorced which is not good”. (W23).

The irritability and emotional exhaustion resulting from the pandemic caused miscarriages and menstrual irregularities among women, in the study area. These experiences worsened the health conditions of these women. Accordingly, respondents explained during the in-depth interviews.

“Yes, I will not lie, this pandemic makes me irritated at any little thing and when I think about it, I feel very emotional and tired of life, but I can’t blame anybody because it just happened. Even my menstrual cycle is now not regular, and this is because of COVID-19”. (W6).

“Now, I am pregnant and fear that I can get miscarriage, but I don’t want to lose my baby because of COVID-19. I had two miscarriages when the pandemic started, because of anxiety. The second was serious so I was admitted in the hospitals for some days because I was bleeding too much”. (W17)

#### Worsened pre-existing health conditions.

Most of the participants had their pre-existing health conditions getting worse, during the lockdown because it was difficult accessing healthcare facilities. Participants attributed this to healthcare workers paying more attention to patients with COVID-19 disease, thereby neglecting patients who reported with other diseases.

“Those with health issues like diabetes nearly died because health workers paid more attention to people with COVID-19. In my case, I went to the hospital some months ago for check-up because I have diabetes and hypertension, but the nurses were busy with COVID patients. Nobody attended to me, so I came home to relax and the next morning I was very sick, so my children rushed me to the hospital and the nurse gave me injection to bring my sugar level down.” (W11)

Few of the women relied on herbal medications instead of replenishing their drugs from the health care facilities and this complicated their health conditions.

“I had diabetes issues because of COVID-19, and I will never forget this experience. My drugs finished but I didn’t go to the hospital for more drugs because the last time I went, nobody attended to me, so I decided to manage the little sore on my leg with local herbs and before I realized, the sore had become very big, and they cut my leg.” (W13)

The health challenges experienced among women during the COVID-19 pandemic were overweight and obesity, fear and anxiety and the feeling of helplessness and insomnia. Other health challenges included depression and worsening of pre-existing health conditions, resulting from the use of herbal medications and limited care form healthcare workers.

### COVID-19 social and health benefits

#### Improvement in health status.

Some of the women improved their diets to stay healthy and to minimize their chances of contracting the COVID-19 virus and dying from the disease, especially, those with underlying health issues, such as, diabetes and hypertension. This has contributed to improving the health of the study participants.

“I will say COVID-19 has kept me on my toes in terms of my health. I now pay more attention to what I eat because I have diabetes and I am scared that if I get COVID, I might not survive it since it has killed my brother. I was obese [obesity] some time ago but now my weight is normal, and this has helped me to maintain my sugar level” (W87).

#### Self-care awareness.

Participants highlighted the important role they play as house managers for the smooth running of their homes. This served as a form of motivation and encouragement to take care of their health by adhering to the COVID-19 protocols, to eat nutritious foods. Staying healthy will enable them to live longer and take care of their families.

“…As for me I follow the COVID-19 protocols to eat more fruits and vegetables so I can be healthy and live longer. I don’t want to die because nobody will take care of my kids. I eat good foods so that even if I get COVID, my system will be strong to fight against the disease”. (W78).

“Before COVID-19, I was eating only carbohydrate foods because these foods are cheap but now, I eat vegetables and other good foods because I don’t want to have diabetes complications and die like my sister, leaving my husband and children”. (W109).

“COVID-19 has taught me the need to eat well and take good care of myself and I will not stop this practice even after COVID-19 because life comes first. As a woman, I want to be strong and take care of my family. You know women are supposed to take care of their homes and protect their families”. (W88)

#### High sense of responsibility towards extended families.

The deadly nature of COVID-19 has contributed to participants increasing their sense of responsibility towards their extended family members. They believed that paying more attention to their family members by encouraging and supporting their feeding could prevent COVID-19 related deaths.

“COVID-19 has increased my desire to pay more attention to my extended family members, so they can live longer. One of my sisters has lost her job so I have been sending her some money for food so that she doesn’t fall sick and die. This is something positive I took from the COVID-19 pandemic”. (W12)

“One good thing about COVID-19 which will stay with me for the rest of my life is the need to be responsible for my extended family members. I call them every day to encourage them not to lose hope in this challenging moment. Every week, I take food to my uncles, brothers, and sisters because I feel I am responsible for them. If I don’t support them and anything bad happens to them, I will feel very guilty”. (W57).

#### Strengthened core family relationships.

Participants explained that the lockdown helped to build strong relationships with their nuclear families. Majority of these women were working from home with their spouses and children during the pandemic. They explained that, before the COVID-19 pandemic, their spouses reported home late after work and their children also went out to play with their friends after school, which hindered them from spending quality time together as a family.

“I am happy because COVID-19 has made us spend more time together as a family because with the lockdown, my husband and children are always home. Before, when my husband closes from work at 5pm, he gets home as late as 10pm because he goes out to drink with his friends leaving me alone in this house. The children also want to be with their friends to play after school. This is good because I have been able to build a strong relationship with my family members because of COVID-19 lockdown”. (W32)

“One thing I like about COVID is that my children now stay home and spend more time with me. They don’t go out to play and come home in the evening as usual. I hope the COVID-19 protocol to stay at home never ends because I am enjoying with my family. I am only sad for those who have lost their jobs and family”. (W18)

Some of the women whose husbands and children lived overseas, could not meet them in person but were able to have frequent communication with them on phone, thereby, binding them together than expected.

“…I have not been able to visit my husband and children in Australia because of COVID-19 lockdown but I talk to them every day because they are working from home, and this has enabled me to build the bond between my husband and children. Before the lockdown, it was difficult spending more time with my husband on phone because he is always busy with work, but you know as a woman I need more attention from my husband. I will say that the lockdown has helped me a lot because my relationship with my husband is better than before.” (W48)

#### Work-family balance.

Many of the study participants identified more benefits associated with working from home. The flexibility associated with working from home enabled them to balance their work and family life, subsequently, increasing work efficiency. This was observed mostly among the married women with children,

“We all know that COVID-19 has created a bad atmosphere for everyone but one of the benefits for workers is the quality time we spend with our families alongside working from home. As a married woman with kids, working from home help me to attend to my family at any time, which increase my work output”. (W39)

“In fact, I didn’t like Covid-19, but I enjoyed working from home. As a lab technician, usually, I come home late because I have a lot to do at work such as running around to make sure the lab is set for experiments and facilitating the purchasing of chemicals and equipment for experiments. Now, I do some paper works from home and spend more time with my kids and husband, which is good”. (W52)

#### Healthy lifestyle behavior.

Some of the study participants explained how COVID-19 has contributed to improving their previous behavior, such as, avoiding alcohol intake and smoking. Those who used to smoke and take excessive alcohol, decided to stop, to improve their health and prevent death.

“We all know that COVID-19 is not a good thing, but it has helped me to change my bad behaviors like drink too much alcohol and smoking. I have stopped because I don’t want to get COVID-19 and die. My doctor has been advising me to stop drinking and smoking and eat well so that I can manage my diabetes, but I didn’t listen. I have stopped now because COVID-19 has killed two of my aunties and I don’t want to follow them to the cemetery. I have even started jogging to improve my health” (W 59)

The social and health benefits of COVID-19 included improvement in health status, self-care awareness, healthy lifestyle behaviors and high sense of responsibilities towards extended families. The bond between core family members were strengthened and those working from home were able to balance their work and family lives.

## Discussion

This study reveals employment and financial issues among women, during the COVID-19 pandemic. Whilst most of them were worried about the possibility of losing their jobs, those who had already lost their jobs, expressed difficulties in getting new jobs. Studies have reported a decline in employment as well as income and job losses, resulting from the economic impact of the COVID-19 pandemic [76,77]. Participants job renewal challenges and fear of losing their job, highlight issues of job insecurity, among women in the study area, which needs to be addressed. The COVID-19 pandemic could have contributed to this, by creating a sense of powerless that resulted from participants perceived threat to their job continuity [78]. Though individuals have different opinions and experiences regarding their job insecurity after been exposed to similar stressful circumstances, its impact is more intense than job loss [79]. Unexpected organizational transformations including layoffs affect women more, since they are already underemployed and underpaid in the labor market [23]. This leads to economic inefficiency, hinders economic growth and the attainment of the sustainable development goal 5 [80]. On the other hand, employers’ ability to improve their retention policies, could lessen the adverse effects of job insecurity among women in their workplaces [81,82]. Given that the study area is a male dominant society [44], addressing unemployment and job insecurity issues among women, could reduce gender inequality, thereby contributing to achieving the Sustainable Development Goal (SGD) 5 [83].

Employment can potentially contribute to fulfillng core needs of human beings. The psychology of working theory indicates the importance of working to fulfill survival need, social connection and self-determination [84,85]. The needs taxonomy was established through the synthesizes of literature on work and well-being as well as building on the transformative contributions of Maslow hierarchy of needs [86]. Fundamental to working is its ability to meet a person’s needs by providing the means for survival [84]. Building on the evolutionary history of working as a means of survival, this study identified working as a way of organising the efforts needed to ensure access to basic necessities of life including food and other core attributes to survival. As such, the unemployment experienced among some of the study participants, prevented them from living their normal lives.

Working creates a platform for people to experience the need for social contact and connection and provide a sense of contributing to the overall social and economic well-being of a community [87]. The COVID-19 pandemic closed the opportunity for people to access and enjoy social world, which could have contributed to the feeling of loneliness, anxiety, depression and worsened health conditions among this study’s participants. Other scholars have reported reduced quality of life for women during the pandemic [88,89]. Another essential need of women is centered on the ability of their work to foster a sense of self-determination such as autonomy and achievement [90]. Apart from the varying capacities of countries to provide resources that support self-determined women’s work lives, social and economic conditions constrain can hinder access to sustainable work [91]. The loss of employment opportunities created by the COVID-19 pandemic, represented a source of existential fear and uncertainty which compounded the intense anxieties about painful and premature deaths that occurred [92].

The findings of this study further identified health challenges such as overweight and obesity among women during the pandemic. COVID-19 pandemic changed the social norms, including restricted access to exercise facilities, working from home, and preventing social gathering [1,79]. The impact of these on weight is crucial, especially for individuals with obesity, by changing their dietary behaviors [93–95]. Most of the women in the study area, used food to cope with COVID-19 related stress and anxiety, thereby increasing their weight. This study adds to knowledge by highlighting the influence of COVID-19 on the dietary behavior of women. It is essential for women to prioritize their weight management to improve their health [96,97].

Additional health challenges including fear, anxiety, and depression with greater impact on women who had experienced quarantine, conforms with other studies [98,99]. Restrictions that prevent individuals from interacting with families, and other people increase anxiety and depression in their day to day lives [100]. Anxiety that results from pregnancy and insecurity connected to natural disasters can disturb the mental health of pregnant women [101]. As such, the miscarriages experienced by the study participants could be due to anxiety from the pregnancy itself and the COVID-19 pandemic. These mental health issues combined with the inability of women to sleep, if not addressed, could worsen their health status, since women are more vulnerable to sleep problems [102]. Previous research on viral outbreaks such as severe acute respiratory syndrome and Ebola identified short- and long-term effects on the mental health of people [103,104]. This calls for appropriate mental health interventions to address the undesirable short- and long-term effects of COVID-19 pandemic, among women, in the study area.

The limited access to health care facilities which worsened the pre-existing health conditions of women in this study, is consistent with the work done by other researchers [105,106]. The COVID-19 pandemic disrupted access to routine medical care, where healthcare workers were assigned to other tasks, and in person appointments considerably reduced [107–109]. Participants’ diabetic foot sore complication and amputations could have been avoided if adequate care was provided by the health care providers, during their initial visits. Since delay in receiving healthcare puts women at higher risk of health complications and death [110,111], appropriate policies are needed to ensure that women in the study area receive adequate health care services, during emergency situations, like the COVID-19 pandemic.

Despite the employment and health issues women encountered during the pandemic, they also identified some social and health benefits, such as, improving their diet to boost their health. Lifestyle changes including positive eating and preparing more meals at home led to shifts in dietary habits, during the COVID-19 pandemic [112,113]. Shifts in dietary habits, where individuals eat nutritious foods, are of great importance in the Fiji Island, where many deaths are attributed to diet and lifestyle diseases like diabetes and hypertension [114–116]. Therefore, strengthening the positive change in dietary behaviors among the study participants with chronic diseases, could improve their health and prolong their lifespan [117,118]. On the contrary, other studies have revealed unhealthy dietary patterns during the pandemic, due to limited financial resources to purchase quality foods [119–121].

Additionally, participants avoiding alcohol consumption and smoking, during the COVID-19 pandemic, provide an opening to intensify education on healthy behaviors, in the study area. The link between chronic diseases and unhealthy behaviors including poor nutrition, high intake of alcohol and smoking has been established [122,123]. Hence, encouraging and strengthening the positive behavioral changes exhibited by women in the study area, could contribute to the management of chronic diseases and prevention of death. Recent review of Terror Management Theory has revealed that death reminders increase commitment of a person’s worldview and threat to a person’s worldview increase accessibility to death-related thoughts [124–126]. Relating this theory to health-related behavior, thoughts of death can either incease motivation for healthy behavior or denial and avoidance when people are consciously focusing on them. In this study, women adapted healthy lifestyle behaviors to live longer and prevent COVID-19 related deaths.

Self-care awareness developed among the study participants, to stay healthy and take care of their families, is a good initiative to promote their health and prevent diseases. This highlights the attitude of women to sometimes internalize societal expectations by caring for others over self [127]. The seemingly superwoman role to assist other people before oneself, to avoid being categorized as a helpless person, could reduce the possibility of women to seek expert care or social support for their mental and physical health, in a timely way [128]. Nevertheless, self-care awareness enables individuals to better recognize their needs, wants, goals, weaknesses, and emotions which could contribute to improving their health [129]. Since most women in the study area, are usually in-charge of the kitchen, understanding the need to eat nutritious foods, would not only improve their health but that of their family members [130].

The high sense of responsibility exhibited by participants towards their extended family members, during the pandemic, strengthened their relationships and bonds. Being responsible for one another is a good thing since it increases a sense of belonging, which could improve the health and well-being of individuals [131]. Participants identified the COVID-19 movement restrictions as an opportunity to spend quality time with their nuclear family. The emphasis of wishing for a prolonged curfew expressed as *“I wish the curfew continues for another year because my husband is always home to spend time with me”,* highlights the important role the COVID-19 pandemic preventive strategies played in strengthening relationships, among core family members. Nonetheless, limiting face-to-face interactions to core network members such as spouses, nuclear family members and possibly individuals living in the same rooms, could lead to the loss of distant relationships, thereby, missing out on diversity of support, opinions, and resources [132,133].

Furthermore, the flexibility of working from home during the pandemic enabled participants to attend to their families at any time, thereby, improving their work and family lives. In a family-oriented community like Fiji, where women have double roles of building their career alongside managing their homes, work-family balance is essential in creating harmony, building a healthy home, and improving work efficiency [134,135]. Time balance is sustained when individuals feel they are spending appropriate amount of time at work and the right amount of time with their families and other social commitments. Therefore, when searching for a balance, the different aspects of life should complement each other, and this could increase life satisfaction [23]. Our findings contradict other studies that identified work-family balance as challenging for women, during the COVID-19 pandemic, subsequently forcing many to restructure their lives [136,137].

This study highlights the importance of understanding employment and financial issues, and health challenges women faced during the COVID-19 pandemic, for suitable interventions. Employers may use these findings to address job and financial concerns raised by women including low salaries and contract renewal issues. Health challenges such as insomnia, obesity, and depression, could have both short-term and long-term effects on women, if not adequately addressed. Therefore, health managers, healthcare workers and policy makers, may use the findings to provide appropriate strategies or interventions to tackle these health challenges. The social and health benefits identified by the women provide an opening to strengthen healthy lifestyle behaviors in the study area.

The study used qualitative methods and as such the findings cannot be generalized. This is because people’s experiences and perspectives vary over time and from one locality to another. Nevertheless, as the first study to be conducted in the study area, it has provided relevant baseline information on the social and health benefits of the COVID-19 pandemic, health challenges as well as employment and financial issues women faced during the COVID-19 pandemic, in Fiji.

## Conclusion

This study has provided significant understanding of the employment and financial state of women, during the pandemic, thereby contributing to the formulation of evidence-based employment policies that are responsive to women’s socioeconomic needs during public health emergencies like the COVID-19 pandemic. Particularly, job policies should address job challenges related to job insecurity, including contract non-renewal and low renumeration. Providing accessible unemployment benefits is essential to support women facing economic hardship during times of crisis. Furthermore, sponsorship schemes and education loans should be available for women who require additional training to secure their jobs, as skills upgrading can improve job security and mitigate the impact of low salaries.

Additionally, targeted interventions are needed to address the health-related challenges women experienced during the pandemic. Health managers, healthcare workers and community leaders should collaborate to implement strategies that reduce the risk of long-term health complications. Women must be educated on managing chronic conditions and on the potential risks associated with the unsupervised use of herbal medicines. To ensure continuity of care, specialized healthcare services should be provided for women with pre-existing medical conditions during emergencies. Promoting healthy lifestyle behaviors like avoiding alcohol consumption and smoking, and balancing work and family life, is crucial to improve the health and well-being of women. Strengthening family support networks may also serve as a protective factor against the psychological effects of depression, loneliness and helpless feelings experienced among the study participants.

The findings of this study provide a baseline information for further research and underscore the need for interventional studies aimed at improving women’s mental and physical health, as well as their access to healthcare during emergency situations like the COVID-19 pandemic. Targeted interventions could focus on using family support groups for mental health and educational campaigns to promote healthy lifestyle choices identified in this study.

## Supporting information

S1 File Semi-structured interview guide. (DOCX)

S2 File Thematic mapping diagram. (PDF)

S3 File Supporting document. (PDF)

S4 File Supporting document. (PDF)
