# Supplementary material for: Benefits and challenges: Qualitative exploration of women’s experiences during the COVID-19 pandemic in Fiji
Source: PLoS One. 2025 Sep 4;20(9):e0331794. doi: 10.1371/journal.pone.0331794 (PMC12410761; doi:10.1371/journal.pone.0331794)
Supplement: S1 File — (DOCX) [file pone.0331794.s001.docx]

**Semi-structured interview guide -** (Expected time duration—30 minutes)

**Demographic Characteristics**

1. Respondent’s unique identification number:
2. Age (completed years):
3. Gender:
4. Tribe:
5. Religion:
6. Marital status:
7. Occupation:
8. Number of children:
9. Study settings:
10. Interview start time:
11. Interview end time:
12. Interview date

**Interview Comments:** This includes recording where the interview took place, mode of respondent during the interview, interactions and other non-verbal expressions of respondents that will help to understand the context of the interview.

**Questions/Probes**

1. Has Covid-19 affected your health and wellbeing in anyway? If yes, in what way? If no, why? *(If not mentioned, probe to find out about issues related to depression, anxiety, loneliness)*.
2. Is there any health issue you experienced during the COVID-19 pandemic? How did you overcome this?
3. Where you able to access health services during the COVID-19 pandemic? If yes, why, if no, why?
4. Can you tell me about some of the health-related challenges you faced during the COVID-19 pandemic? If yes, how did you overcome these challenges?
5. Has Covid-19 affected your social life in anyway? *(If no, why and if yes, why?).*
6. Has Covid-19 affected your employment status and why *(If not mentioned, probe to find out the type of job and if respondent is currently employed).*
7. What job were you doing before the COVID-19 pandemic? Are you still doing the same job? If yes or no, why? *(If the respondent lost her job during the COVID-19 pandemic, ask how she/he sustained or is currently sustaining herself).*
8. Do you think Covid-19 has in anyway affected your livelihood/income and why? *(If not mentioned, probe to find out if Covid-19 affected respondent’s finances).*

Thank you for your time. Please do you have any questions?
