# Supplementary figures and images for: Benefits and challenges: Qualitative exploration of women’s experiences during the COVID-19 pandemic in Fiji
Source: PLoS One. 2025 Sep 4;20(9):e0331794. doi: 10.1371/journal.pone.0331794 (PMC12410761; doi:10.1371/journal.pone.0331794)

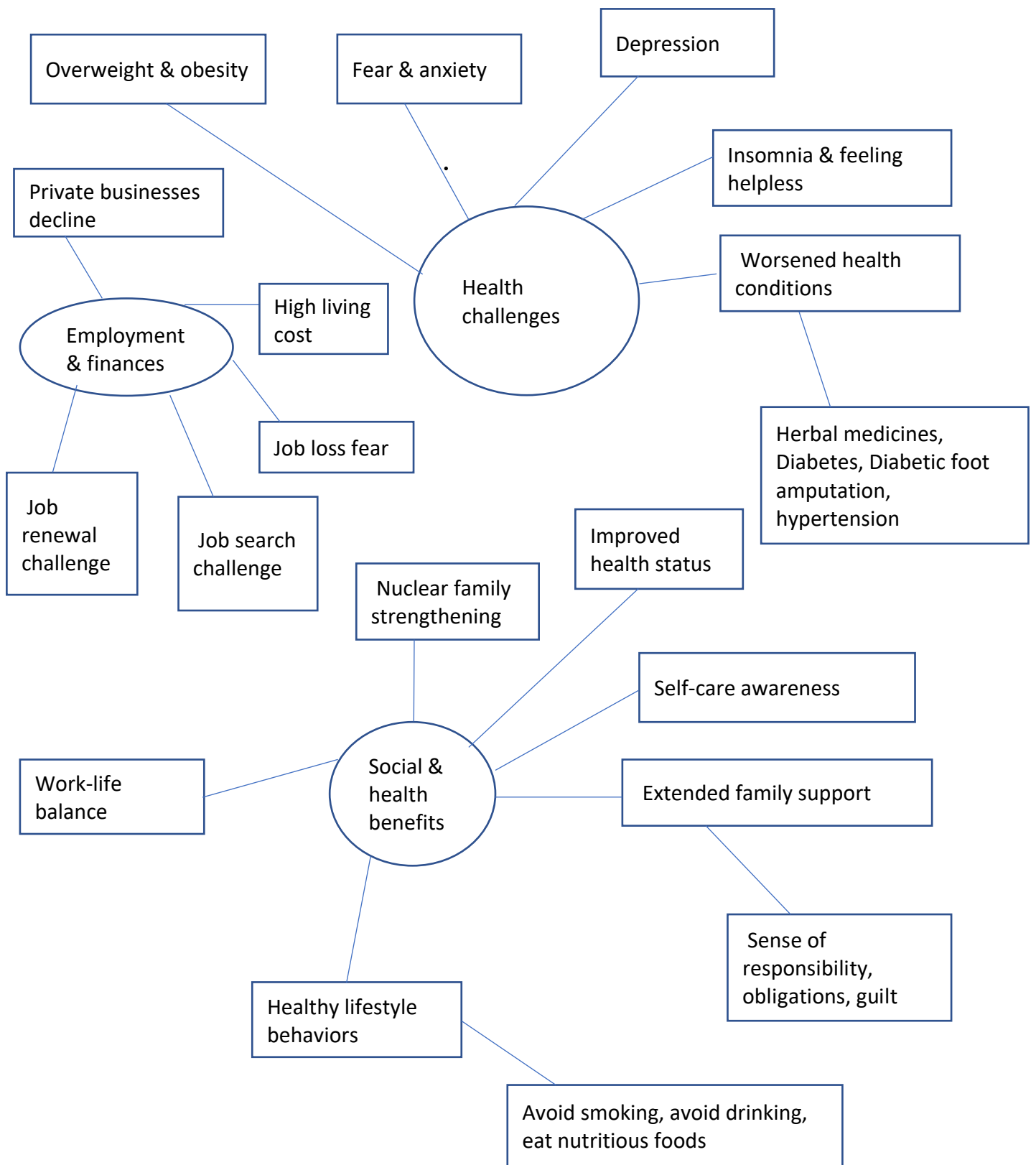

Supplement: S2 File — (PDF) [file pone.0331794.s002.pdf]
