# Supplementary material for: Benefits and challenges: Qualitative exploration of women’s experiences during the COVID-19 pandemic in Fiji
Source: PLoS One. 2025 Sep 4;20(9):e0331794. doi: 10.1371/journal.pone.0331794 (PMC12410761; doi:10.1371/journal.pone.0331794)
Supplement: S3 File — (PDF) [file pone.0331794.s003.pdf]

Interviewer (I): Can you explain to me if Covid-19 has affected your health in anyway?

W2: COVID-19 did not help at all because I was eating too much, and I have put on too much weight during the lockdown. I have become obese, and I think I need to start checking my weight now because I don't want to get diabetes and other diseases.

I: Did you experience health challenges during Covid-19 pandemic?

W102: hmm … In fact we all know that COVID-19 was a big issues because my health has become worse and I don't know if it will get better again. Can you believe I am now on hypertension drugs and this disease will always be there, hmmm not east at all.

I: Kindly explain to me if you experienced any health challenge during the Covid-19 pandemic. Please tell me your experiences.

W74: I was slim before the pandemic, but I have now become big because I eat a lot. My problem is I eat to relax and to take my mind off COVID-19 issues because the anxiety is too much. Anytime I get scared, I find some food to eat and become happy. This is what is keeping me alive but I'm gaining too much weight. Last week, I felt some heaviness in my chest and when I went to the hospital, I nurse told me to reduce my weight because I am overweight. I don't want to become obese [obesity] and get diabetes like my other family members.

I: Have you encountered any health issues due to COVID-19 pandemic?

W32: We are all scared of death, so COVID-19 is a problem for everybody. The cases are going down, but I am still afraid because anything can happen to anybody at any time. I have taken the vaccine, but I don't think it can help so I am scared and feel stressed every day thinking about this pandemic.

I: Any other thing you want to discuss?

W83: Yeah…. Ehmmm… My mental health state became worse after quarantine because I felt lonely in my isolation room and became too anxious that anything can happen to me. Hearing

about COVID-19 deaths stressed me and made me confused. I don’t even know if I can get back to my normal mental state.

I: Can you tell me about any mental health issues you faced?

W90: The truth is I’m not able to sleep at all because of COVID-19 pandemic. COVID-19 information is very scary, and we know some are not true but still I can’t stop thinking about them. The media reports new things every day and this has affected my sleep badly. Do you know that my BP [blood pressure] has gone up so I am taking BP medications now?

I: You have mentioned that you didn’t know what to do during the pandemic. Can you explain further?

W15: I feel everything has come to an end for me because I have lost my job, and I don’t have enough money to even feed my children. I don’t think anybody can help in this situation because everybody is fighting COVID-19 problems. I stay awake every day and it’s affecting my health.

I: Any other issues you want to talk about regarding health during COVID-19?

W1: I went through a state of depression, and I am still fighting with it, because I lost one of my immediate family members because of COVID. There is no way you can stop mourning over losing a family member so anytime you remember, it will push you into a state of depression and fear.

W44: COVID-19 had made me depressed and I’m now taking medications because my dad died in another town, and I couldn’t even see his dead body. My two uncles also died from the pandemic, and I couldn’t attend their funerals. I always feel so guilty because I feel I didn’t protect them enough and that is why they died. I don’t think I can ever forget this in my life.

I: Do you think your health was affected during the pandemic?

W23: yes, hmmmm, it’s a big issue … truth is I have gone through too much depression because of the pandemic. All my plans have come to a halt, and I don’t know what the future will bring

to me. Another thing is that my partner has been away from me due to the lockdown, and we were fighting on phone every day and you know this is depressing. Now we are divorced which is not good.

I: Do you have any other thing to talk about?

W6: Yes, I will not lie, this pandemic makes me irritated at any little thing and when I think about it, I feel very emotional and tired of life, but I can't blame anybody because it just happened. Even my menstrual cycle is now not regular, and this is because of COVID-19.

I: Has Covid-19 pandemic affected your health in anyway?

W17: Now, I am pregnant and fear that I can get miscarriage, but I don't want to lose my baby because of COVID-19. I have had two miscarriages when the pandemic started, because of depression and anxiety. The second was serious so I was admitted in the hospitals for some days because I was bleeding too much.

I: Did you face any health issues during the lockdown?

W11: We those with health issues like diabetes nearly died because health workers paid more attention to people with COVID-19. In my case, I went to the hospital some months ago for check-up because I have diabetes and hypertension, but the nurses were busy with COVID patients. Nobody attended to me, so I came home to relax and the next morning I was very sick, so my children rush me to the hospital and the nurse gave me an injection to bring my sugar level down.

I: Where you able to access health services during the Covid-19 pandemic?

W13: I had diabetes issues because of COVID-19, and I will never forget this experience. My drugs finished but I didn't go to the hospital for more drugs because the last time I went, nobody attended to me, so I decided to manage the little sore on my leg with local herbs and before I realized, the sore has become very big, and they cut my leg.

I: Do you have other experiences during the pandemic that you want to share with me?

W87: I will say COVID-19 has kept me on my toes in terms of my health. I now pay more attention to what I eat because I have diabetes so I am scared that if I get COVID, I might not survive it because it has killed my brother. I was obese some time ago but now my weight is normal, and this has helped me to maintain my sugar level.

I: Are you saying that you experienced some positive things during the pandemic?

W78: I will say, everything is not bad from my side …As for me I follow the COVID-19 safe protocols to eat more fruits and vegetables so I can be healthy and live longer. I don’t want to die because nobody will take care of my kids. I eat good foods so that even if I get COVID, my system will be strong to fight against the diseases.

I: Any behavior changes during the pandemic?

W88: Before COVID-19, I was eating only carbohydrate foods because these foods are cheap but now, I eat vegetables and other good foods because I don’t want to have diabetes complications and die like my sister, leaving my husband and children behind. COVID-19 has taught me the need to eat well and take good care of myself and I will not stop this practice even after COVID-19 because life comes first. As a woman, I want to be strong and take care of my family. You know women are supposed to take care of their homes and protect their families.

I: What about your family life, has anything changed because of the pandemic?

W12: COVID-19 has increased my desire to pay more attention to my extended family members, so they can live longer. One of my sisters has lost her job so I have been sending her some money for food so that she doesn’t fall sick and die. This is something positive I took from COVID-19 pandemic.

I: Can you tell me about the effects of Covid-19 on your social life?

W57: One good thing about COVID-19 which will stay with me for the rest of my life is the need to be responsible for my extended family members. I call them every day to encourage them not to lose hope in this challenging moment. Every week, I take food to my uncles,

brothers, and sisters because I feel I am responsible for them. If I don't support them and anything bad happens to them, I will feel very guilty.

I: Apart from what you have explained, did you experience any good thing during the pandemic?

W32: I am happy because COVID-19 has made us spend more time together as a family because with the lockdown my husband and children are always home. Before, when my husband closes from work at 5pm, he gets home as late as 10pm because he goes out to drink with his friends leaving me alone in this house. The children also want to be with their friends to play after school. This is good because I have been able to build a strong relationship with my family members because of COVID-19 lockdown.

I: What about your family life, do you think Covid-19 affected any aspect of it?

W18: One thing I like about COVID is that my children now stay home and spend more time with me. They don't go out to play and come home in the evening as usual. I hope the COVID-19 protocol to stay at home never ends because I am enjoying with my family. I am only sad for those who have lost their jobs and the deaths occurring.

I: Please explain to me how Covid-19 has affected your social life.

W48: hmmm Oh yes …I have not been able to visit my husband and children in Australia because of COVID-19 lockdown but I talk to them every day because they are working from home, and this has enabled me to build the bond between my husband and children. Before the lockdown, it was difficult spending more time with my husband on phone because he is always busy with work, but you know as a woman I need more attention from my husband. I will say that the lockdown has helped me a lot because my relationship with my husband is better than before.

I: What about trying to combine the work you do and taking care of your family. Did Covid-19 affect these in anyway?

W39: We all know that COVID-19 has created a bad atmosphere for everyone but one of the benefits for workers is the quality time we spend with our families alongside working from home. As a married woman with kids, working from home help me to attend to my family at any time, which increase my work output.

I: Do you have any other thing to talk about?

W52: In fact, I don't like Covid-19 but I enjoy working from home. As a lab technician, usually, I come home late because I have a lot to do at work such as running around to make sure the lab is set for experiments and facilitating the purchasing of chemicals and equipment for experiments. Now I do some paper works from home and have more time for my kids and husband which is good.

I: What are the issues you faced during the pandemic that you can share with me?

W59: We all know that COVID-19 is not a good thing, but it has helped me to change my bad behaviors like drink too much alcohol and smoking. I have stopped because I don't want to get COVID-19 and die. My doctor has been advising me to stop drinking and smoking and eat well so that I can manage my diabetes, but I didn't listen. I have stopped now because COVID-19 has killed two of my aunties and I don't want to follow them to the cemetery. I have even started jogging to improve my health.

I: Has Covid-19 affected your employment status?

W4: To be frank my biggest fear is losing my job since this has become the order of the day. Even two of my cousins and some friends have lost their jobs because of COVID-19. Our economy is going down, so anything is possible.

I: Can you explain further about what the problem is?

W2: I am worried because I think from the way things are going, my employers can terminate my job at any time. The last time they sacked almost all the cleaners and brought new ones so it can happen to me too.

I: Now that you have lost your job because of Covid-19, have you made any attempt to find another job?

W7: I lost my job because of COVID, and I have started looking for a job that can fetch me some money to sustain myself and my family, but it seems that is not possible now. I saved some money from my previous work, but that money is almost finished because I used some to buy food, pay rent and pay bills. It is a big problem but there is nothing I can do.

I: Can you explain to me if Covid-19 has affected your job?

W70: I am sad…I have lost my job, and I don't have any money saved in my bank account to cater for my children. I could not save any money because my salary was not even up to 1000 dollars a month and I have children to cater for. I am a single mother with 6 children so you can imagine what I am going through financially.

I: Are you saying that you have some obligations to meet to renew your job contract?

W10: Oh yes and Covid-19 has made matters worse…I have received a letter from HR [human resource] to upgrade our qualification, else I will lose my job but it's not easy to learn and go through COVID-19 stress at the same time. I have enrolled for some online courses to upgrade my qualification and keep my job, but I cannot concentrate.

I: In what way has Covid-19 affected your employment status?

W15: I must upgrade myself to maintain my job, but the problem is, nobody is paying the school fees. I have to pay my fees from my salary which is not enough. The best thing for me to do now is to look for a new job but I don't think I will get one because COVID-19 has affected the economy. I have no option than to upgrade my skills to keep my job.

I: What can you say about other businesses. Has Covid-19 affected the operation of your own businesses?

W90: I am not a government worker. I buy fish from fishermen and sell in small quantities in the market, but everything has come to an end because of COVID-19. Fishermen have been

banned from going to the sea to fish, so the business has gone down. I know the little money I have will soon finish because things are expensive now.

I: Do you think Covid-19 has affected your income in anyway?

W85: I was making a lot of money from sewing. I have worked as a seamstress all my life, but I have to close my shop because of COVID-19. My sewing business is going down every day because people don't have money to sew dresses. Only few family members bring their materials for sewing and they don't even pay for the sewing because they don't have money. It's a big problem but I pray things get better soon.

I: Please explain to me how Covid-19 has affected your livelihood.

W19: In fact, the prices of things have gone up. Even food is expensive because of COVID-19…At first, I was able to save some money from my monthly salary but now I cannot do that because things are very expensive, but the government has not increased my salary.

W110: hmmm, ehmmm for me all the money I saved is finished because the prices of things have gone up and I have lost my job because of COVID-19.
