# Supplementary material for: Benefits and challenges: Qualitative exploration of women’s experiences during the COVID-19 pandemic in Fiji
Source: PLoS One. 2025 Sep 4;20(9):e0331794. doi: 10.1371/journal.pone.0331794 (PMC12410761; doi:10.1371/journal.pone.0331794)
Supplement: S4 File — (PDF) [file pone.0331794.s004.pdf]

I: Are you saying covid-19 influenced your life in a good way?

W101: yes, covid-19 has encouraged me to take my health issues serious to stay healthy and support my family. I'm diabetic so I check my sugar level regularly to prevent diabetes complication because my brother had his foot amputated from diabetes. Sometimes I walk around the house to keep my system active. Covid is bad but I have benefited in some ways and will continue even after the pandemic.

I: Can you tell me more about your experiences during the pandemic?

W86: The truth is, I'm always afraid of getting covid-19 because I don't want to die and leave my children in this world alone. I'm always panicking because I don't know what the future holds for me since covid-19 has changed everything now.

I: Don't you think you should seek for help?

W33: I'm very sad and I don't think anybody can help me in this situation. Three of my relatives have died from covid and I'm worried because I can also die. I stay awake every night crying and I don't know when this will end.

I: What about your social life?

W72: covid-19 has brought loneliness to me and I know some people are also going through the same thing which comes with too much sadness. I think social media is not helping because they talk about things which are not true, just to put fear in us. I have not been able to sleep well for months, and it has made my blood pressure gone up.

I: So, what did you do. Did you go to the hospital for treatment?

W108: The last time I had severe chest and neck pains and that almost took my life. I started using herbs when my dedications got finished and ended up with serious complications. covid-19 has put too much fear in people and I feel very lonely all the time. It's also due to the fake news the media has been reporting every day. I stay in my house all the time instead of going out to stretch my body and exercise to keep my sugar under control.

I: Was your job affected in any way?

W14: hmmmm I don't want to remember this pandemic because I have lost my job. My husband also lost his job due to covid-19, so we don't have enough money to support our family. This is stressing me because the little savings we have is almost finished. Ehmmm now we don't know what the future holds for our family because the economy is going down every day.

I: What employment challenges have you experienced?

W68: covid-19 is not a good thing, since I lost my job, I have not been able to get another job, but I think the problem is everywhere in the world. My cousin in Canada has lost her job and my brother in Australia is also struggling to find another job. Now they only advertise for few jobs for health workers not everybody.

W27: hmmmm ehmmm I was a cleaner but covid-19 made them terminate my contract. My monthly salary was not much so I have finished the little money I saved. I have searched the internet and called friends for another job but there is no job available now for me. I'm frustrated and depressed and on medications because I think too much about the future.

I: Has covid-19 affected your health in any way?

W51: to say the truth, the pandemic has changed my moods, sometimes I'm happy and another time you will see me crying and staying awake throughout the night. I'm in a state of depression and taking medicines to maintain my mental health, else I can go crazy. I started taking medications after covid killed two of my friends and three relatives. Nobody expected them to die because they looked strong.

I: Has anything affected your income level.

W94: yes, you know the economy is gradually collapsing and some of our employers are not able to pay our salaries. I work as a sales manager in a private sector and I'm still waiting to receive my 3 months' salary. I'm using my savings and it's not enough. It's very frustrating because I don't think I can keep my job. My sister lost her job unexpectedly due to covid-19

and she is now depressed. She is not the only person, those who were working in the hotels all lost their jobs.

I: You mentioned that your health has not been the same since the beginning of the pandemic, can you explain further?

W46: oh yes, I was saying that I have bad experiences from the pandemic. I lost my pregnancy, and I always think too much about it that I cannot sleep well. Anytime I think about having another baby, I get scared because I don't want to have another miscarriage and go through the pain again.

I: In what way has covid-19 pandemic affected your wellbeing?

W53: I have been emotionally unstable since the onset of the pandemic. My doctor said I have depression because I cannot eat, sleep or talk to my family. when my mother died of covid, I have not been myself because I feel guilty that I did not do enough to prevent the death. I'm very sad about the situation.

I: What about your family. Is everything ok?

W99: all is not well with me because my relationship has collapse due to covid lockdown. This happened when my boyfriend was transferred to assist covid patients in Nadi and we could not visit each other due to covid lockdown. We were fighting on phone and finally broke up and this affected me mentally.

I: Any social issues you want to talk about?

W9: I enjoyed my husband's company better during the lockdown so in that way I will say covid was good because I have built my relationship and I'm happy. We support ourselves as a family which is good. What I do is to pay attention to my work and when I work for a while I attend to my family.

I: What are the health challenges you faced during the pandemic?

W3:  the problem is my weight because I have put on too much weight. I'm not able to go out for a walk so my sugar level has increased. My friend who is a nurse told me to eat more vegetables but it's very expensive. Covid has made all situations bad, prices of foods and other items have gone up. Even taxi fare has increased so I need financial support because it's not easy.

I: so, if you have taken the vaccine, why are you still afraid?

W80: yes, I have taken the vaccine, but I feel that cannot protect me from covid. I'm scared and don't know what to do.

I: Can you tell me how the covid-19 pandemic affected your social life?

W60: covid-19 has made me so lonely because nobody visits me at home again and this is stressing me. Even now I feel scared and afraid that I can get covid because many people have died from covid. Sometime ago they buried more than 1000 people in America and other countries, which is confusing because how can one virus be killing many people like this. In fact, fear and loneliness is killing me.

I: Please tell me about your covid-19 experiences?

W97: hmmm … emmmh covid is not a good thing because I lost my job and many people in the world also lost their jobs, but it has made me to pay more attention to my health. I now eat good foods and follow the advice from my doctor. I check my sugar level and take my drugs to keep my sugar normal. So, you see covid has taught me good things.

I: Is there any other thing you want to share?

W26: I just want to add that the government should support more women in situations like this so that we can support our family members. People are dying from covid every day, so I always call my parents and sisters to encourage them and share the little money and food I have with them to keep them going. If I don't support and they die, I cannot forgive myself.

I: Did you make any adjustment because of covid-19?

W35: to be frank, I enjoyed working from home because my workload reduced, and my children and husband are always available to assist me at home. We do things together which has improved the bond we share as a family.

I: please share with me some of your experiences during the pandemic?

W105: the lockdown was a big problem for me because I was on my own all the time, so I felt very lonely and was depressed. Anytime I felt unhappy, I will get some food to eat to calm my nerves down. My doctor told me I have become obese so I'm trying to reduce my weight to live long.

I: Do you have anything to add to what you have already explained.

W103: I will say this is a trying moment for everyone, the economic situation is bad in most countries, so it has been difficult for me to get a job. I have not seen any accounting job I can apply for online, except few for health workers which I don't qualify. The more I search, the more I find it difficult to sleep. In fact, I'm tired of life and don't know what to do.

I: What about your health?

W42: as for me the pandemic has affected my sleep, I cannot have a continuous sleep because I wake up in the middle of the night and I find it difficult to sleep. I have been thinking about how to renew my contract and keep my job. I must upgrade my qualification, but the training I need is very expensive and I don't have enough money for that. I think is not fair but if I don't comply, I can lose my job at any time.

I: Have you experienced anything different during the pandemic?

W64: one bad thing about covid-19 is that we are not sure what the future holds because I'm facing financial difficulties since my business collapsed. I was selling ladies dresses and shoes, but people are not buying now because there is no money but one good things about covid-19

is the lockdown. With the lockdown, my husband and children are always home with me and I’m happy. My marriage has become better now so I didn’t want the lock down to end.

I: So, what are you afraid of?

W106: my job is the main thing I’m afraid to loss because if that happens, I cannot even feed myself and my family. People are losing their jobs every day, so I’m scared to go thru that.

I: In what way has covid-19 affected your social life?

W91: before covid-19 you will never see my husband at home. He will be outside with friends and come home very late after work. Covid has taught me how to manage my time well to accomplish my work and enjoy my family alongside.

I: Is there any other thing you want to say?

W26: when I think about the number of people who have lost their jobs, I am not able to sleep. I cannot give you the exact number, but I can say more that 30 people lost their jobs every day when the pandemic started so you can imagine the number of people who will be unemployed in a month. You cannot even count, and this put too much fear in me every day because it can happen to me too.

I: can you share your covid-19 experience with me?

W108: as for me, the sudden deaths due to covid-19 made me depressed when I think too much about it, but I also benefited from covid-19 because I have stopped taking alcohol, I don’t smoke again. I had some bad friends who introduce me to these things some time ago, but I have stopped because of covid. I follow covid protocol and manage my diabetes properly so I can live long and be there for my children. I want to continue these good practices even after covid.

I: what can you say about your relationship situation during the pandemic.

W40: I was very happy with my husband before the pandemic but now we are no more together because we were always fighting. The fight started when I lost my job could not find another job. There is no money to keep us going and as a woman, I have needs. I will say covid destroyed my marriage because we could not manage the situation properly. I could not communicate well with my husband to keep things under control, but I also blame it on the bad economic situation. Covid-19 has destroyed everything.

I: what are some of your covid-19 experiences?

W82: Ehmm I have both good and bad experiences. What I don't want to remember is covid-19 deaths but I'm able to support my extended family members with food and some money because I have not lost my job like my sister. I call them every day to be sure they are doing well which is good because we have to support our families all the time, it's our culture.

I: Any other thing you want to discuss?

W66: my main issue with covid-19 is people losing their jobs without enough savings to cater for their families. I have closed my shop for a long time now because of covid so I don't have customers again to buy my things which is not good. Ehmmm but I still have to thank God because covid has made me to pay more attention to my health, so I don't die.

I: Did you say you now take care of your health better than before?

W16: yes, I think that is the good thing about covid-19 because nobody wants to die so I try my best to follow covid-19 protocols, I also eat more fruits and vegetables. This will help me to live long to take care of myself and my family because they all look up to me for support.

I: Did the lockdown affect your life in anyway?

W37: I will say yes but in a good way. I enjoyed the lockdown because as I work from home, I'm able to take time off work and attend to my children and husband so I spent good time with my family members.

I: were you able to access health services during the covid-19 pandemic?

W80: it was not easy at all, I have diabetes and because the health workers were paying more attention to covid patients, I had complications, my BP and sugar went up. Next time they should reserve some clinics for us not only covid patients.

I: can you share some of your experiences during the pandemic with me?

W77: yes, covid has helped me a lot because before covid I didn't care about my health. I was eating anything and drinking too much kava but now I don't because I want my system to be strong to fight covid-19. I don't want to die young. I have my sisters, children and other family members to support.
